# Differential Expression of miRNAs in Colorectal Cancer: Comparison of Paired Tumor Tissue and Adjacent Normal Mucosa Using High-Throughput Sequencing

**DOI:** 10.1371/journal.pone.0034150

**Published:** 2012-04-17

**Authors:** Julian Hamfjord, Astrid M. Stangeland, Timothy Hughes, Martina L. Skrede, Kjell M. Tveit, Tone Ikdahl, Elin H. Kure

**Affiliations:** 1 Department of Genetics, Institute for Cancer Research, Oslo University Hospital, Oslo, Norway; 2 Department of Medical Genetics, Oslo University Hospital, Oslo, Norway; 3 Department of Oncology, Oslo University Hospital, Oslo, Norway; 4 Department of Pathology, Oslo University Hospital, Oslo, Norway; 5 Faculty of Medicine, University of Oslo, Oslo, Norway; 6 Department of Environmental and Health Studies, Faculty of Arts and Sciences, Telemark University College, Bø, Norway; Queen Elizabeth Hospital, Hong Kong

## Abstract

We present the results of a global study of dysregulated miRNAs in paired samples of normal mucosa and tumor from eight patients with colorectal cancer. Although there is existing data of miRNA contribution to colorectal tumorigenesis, these studies are typically small to medium scale studies of cell lines or non-paired tumor samples. The present study is to our knowledge unique in two respects. Firstly, the normal and adjacent tumor tissue samples are paired, thus taking into account the baseline differences between individuals when testing for differential expression. Secondly, we use high-throughput sequencing, thus enabling a comprehensive survey of all miRNAs expressed in the tissues. We use Illumina sequencing technology to perform sequencing and two different tools to statistically test for differences in read counts per gene between samples: edgeR when using the pair information and DESeq when ignoring this information, i.e., treating tumor and normal samples as independent groups. We identify 37 miRNAs that are significantly dysregulated in both statistical approaches, 19 down-regulated and 18 up-regulated. Some of these miRNAs are previously published as potential regulators in colorectal adenocarcinomas such as miR-1, miR-96 and miR-145. Our comprehensive survey of differentially expressed miRNAs thus confirms some existing findings. We have also discovered 16 dysregulated miRNAs, which to our knowledge have not previously been associated with colorectal carcinogenesis: the following significantly down-regulated miR-490-3p, -628-3p/-5p, -1297, -3151, -3163, -3622a-5p, -3656 and the up-regulated miR-105, -549, -1269, -1827, -3144-3p, -3177, -3180-3p, -4326. Although the study is preliminary with only eight patients included, we believe the results add to the present knowledge on miRNA dysregulation in colorectal carcinogenesis. As such the results would serve as a robust training set for validation of potential biomarkers in a larger cohort study. Finally, we also present data supporting the hypothesis that there are differences in miRNA expression between adenocarcinomas and neuroendocrine tumors of the colon.

## Introduction

Colorectal cancer (CRC) is one of the most frequently occurring cancers worldwide [1]. Prognosis depends on tumor stage at the time of diagnosis. There is high focus on discovery and validation of early detection markers as well as on predictive and prognostic factors as reviewed by Asghar *et al*. [2]. The molecular genesis of CRC is among the best described of all human cancers. The Vogelstein model [3] has over the years been modified and extended, as exemplified by Slaby *et al*. [4].

MicroRNAs (miRs) are small non-coding RNA molecules 18-25 nucleotides in length, first discovered in the early 1990s in *C. elegans* [5]. They maintain homeostasis by altering gene expression in different cell processes such as differentiation, proliferation, survival and apoptosis [6]. It is estimated that more than 10% of all protein-encoding human genes may be regulated by these mechanisms [7]. The latest number of human miRs recorded in miRBase exceeds a thousand [8], and the increasing use of high-throughput sequencing is driving further discovery. Studies have also shown that miRs may be dysregulated in different human cancers, and hence act as tumor suppressors or oncogenes [9], [10]. These molecules are interesting since they may be potential biomarkers of diagnosis or prognosis and act as potential targets in cancer specific therapy as reviewed by Cho *et al*. [11], [12]. The ultimate goal would be personalized medicine with genotype-phenotype cancer networks as the roadmap to clinical decisions [13].

Many studies have focused on miR expression profiling in colorectal cancer. Most of these studies have analyzed a smaller number of miRs using real-time polymerase chain reaction (PCR) or hybridization based technology, partly from cell lines or non-paired patient tissues [14], [15], [16], [17], [18]. Only a few studies have more globally sequenced miRs in a larger scale for the expression profile, like the study on the melanoma [19] and colorectal [20] microRNAome. The latter study was unique in its kind and presented a set of novel putative miRs by using an experimental approach named miRAGE. However, as this study dates back several years, only a subset of mature miRs known today was actively investigated.

Global expression of miRs has traditionally been assessed using hybridization based array technologies. These arrays are based on sequence specific hybridization after labeling with a fluorescent dye. Fluorescence intensity is recorded and reflects the expression of a given gene. By using multiple dyes, the difference in fluorescence may be used as an index of gene expression. High-throughput sequencing, on the other hand, uses sample transcripts as starting template. Direct sequencing is then performed with a series of reactions using fluorophore terminator nucleotides. Sequence reads are then mapped back to the reference genome or a database of transcripts and the number of sequence reads mapping back to a specific transcript is a measure of gene expression. In the general case of mRNA, this count needs to be normalized for the length of the transcript and the total number of reads generated for the sample. In the case of miR, the normalization for the transcript length is not required as the reads cover the full-length of the transcript. Differential expression is then measured by the difference in normalized counts for a given gene. A recent publication compares differential gene expression in *D. pseudoobscura* when using array technology and high-throughput sequencing. The majority of expression levels are similar between the methods with a comparable performance [21]. A similar study on *S. cerevisiae* has shown that the methods agree fairly well for genes with medium levels of expression, but correlation is very low for genes with either low or high expression levels. This is partly due to the greatly increased dynamic range for quantification of gene expression provided by the high-throughput sequencing method [22]. High-throughput sequencing is further considered superior when dealing with the structure and dynamics of the transcriptome. Examples of this include expression of unknown target sequences, RNA editing events and other RNA sequence variations such as polymorphisms [21], [22], [23].

Since these features of high-throughput sequencing suggest that it is an excellent method for global surveys of small RNAs, we included eight patients with colorectal cancer undergoing surgical resection of the colon for studying tumor specific changes in miR expression using Illumina high-throughput sequencing technology. Tissues of normal mucosa and tumor were collected from surgical specimens for all patients, hence yielding a unique set of paired samples. Our analysis of the sequence datasets we produced from these samples enables us to identify miRs that have not previously been associated with colorectal adenocarcinomas. We have also identified differences in miR expression between adenocarcinomas and a neuroendocrine tumor of the colon. These results add to the present knowledge on miR dysregulation in colorectal carcinogenesis.

## Results

Eight patients were randomly selected according to gender specifications (males only) from a colorectal cancer cohort. Total RNA from tumor tissue and adjacent normal mucosa was extracted. In preliminary analysis of differential expression between tumor and adjacent normal mucosa, one pair demonstrated an expression pattern different from the rest of the pairs. Histopathology was reviewed by a pathologist (Table 1), and it was evident that one patient was misclassified and harbored an atypical neuroendocrine tumor (NET) whereas the rest were adenocarcinomas. All further statistical analyses treated the patient with NET as one separate case from the remaining patients. The percentages of tumor cells and stromal components were also estimated in hematoxylin and eosin stained sections from primary tumor, showing that seven of eight samples harbored more than 60% tumor cells (Table 1).

**Table 1:** Clinical and histopathological characteristics of patients in the study.

Patient ID	Age	Gender	Histology	Differentiation	TNM classification	Anatomic site	Est’d percentage tumor/stoma
1	56	Male	Neuroendocrine	-	T3 N3 Mx	Coecum	85/15
2	71	Male	Adenocarcinoma	Moderate	T3 N0 Mx	Rectum	60/40
3	79	Male	Adenocarcinoma	Moderate	T2 N0 Mx	Coecum	80/20
4	62	Male	Adenocarcinoma	Moderate	T3 N0 Mx	Rectum	10/90
5	55	Male	Adenocarcinoma	Moderate	T3 N0 Mx	Sigmoid	65/35
6	49	Male	Adenocarcinoma	Moderate	T3 N0 Mx	Sigmoid	70/30
7	66	Male	Adenocarcinoma	Moderate	T3 N2 Mx	Rectum	75/25
8	44	Male	Adenocarcinoma	High	T2 N0 Mx	Rectum	90/10

**Figure 1:**
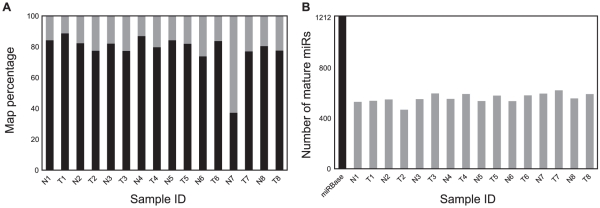
Read classification as predicted by miRanalyzer and miRBase. Panel A with percentage of sequencing reads mapped to mature miRs (black) of the total reads per experiment. Panel B with number of mature miRs identified per sequencing experiment. The total number of mature human miRs in miRBase release 16 (n = 1212) is included as reference.

The 16 samples were successfully sequenced using Illumina Genome Analyzer II (Illumina, CA, USA) and processed using miRanalyzer [24] with an average of 562 mature miRs mapped to miRBase per sequencing experiment when permitting one mismatch nucleotide (Figure 1B). Approximately 80% of sequencing reads mapped to mature human miRs in miRBase (release 16) in seventeen of eighteen sequencing runs, the remaining reads mostly map to other parts of the transcriptome. In the last sample (normal tissue N7) there was a much lower percentage of reads that map to miRBase (37.2% of the total reads) (Figure 1A). This may be due to technical issues during sample preparation. Furthermore, a few hundred putative novel miR sequences and gene loci in the reference genome (hsa hg18) were predicted from the sequencing runs. These putative sequences amount to a small fraction of the total read count (data not shown).

**Figure 2:**
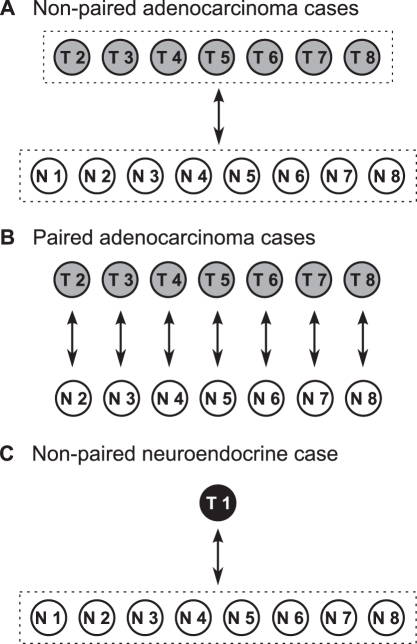
**Schematic illustration of statistical approach.** Panels A and C show approach using non-paired statistics and the DESeq tool. Panel B shows approach using paired statistics and the edgeR tool. See text for further details.

Differential expression (DE) of identified miRs from miRBase was calculated using two bioinformatic tools, DESeq [25] and edgeR [26]. EdgeR implements functionality to perform both paired and non-paired tests (the pair information is ignored, and normal and tumor samples are treated as independent groups), whereas DESeq cannot perform paired tests, but benefits from additional statistical refinements relative to edgeR. Treating the normal and tumor samples as two independent groups is theoretically predicted to be the more conservative test since, unlike the paired test, it does not account for baseline differences between patients. By using both methods, we get two sets of significantly differentially expressed miRs. The intersection between these two sets is a very conservative prediction of the significantly dysregulated miRs. In addition, we were able to observe to what extent the non-paired testing is more conservative than the paired. First fold change of known miRs was analyzed between the groups of adenocarcinoma (n = 7) and normal mucosa (n = 8), subsequently between the neuroendocrine case (n = 1) and normal mucosa (n = 8) using DESeq (Figures 2A and 2C). When looking at the adenocarcinomas as a group and using the Benjamini and Hochberg adjustment [27] for multiple testing (FDR < 0.1), a total of 52 miRs were significantly dysregulated compared to that of the normal mucosa: 28 were down-regulated and 24 up-regulated (Table S1). The neuroendocrine case, however, demonstrated a total of 38 miRs significantly dysregulated compared to the normal mucosa group, all up-regulated (Table S2). Interestingly, only 6 miRs are represented in both histopathological groups: miR-7, -96, -204, -1269, -1827 and -3177. In this analysis there are hence a total of 46 and 32 miRs that seem somewhat specific to the adenocarcinoma and neuroendocrine histopathology, respectively.

**Figure 3:**
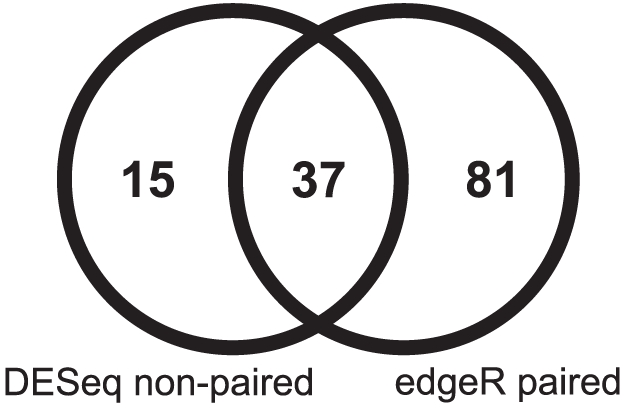
Venn diagram shows the number of significant miRs identified using the non-paired (DESeq) and paired (edgeR) analysis approach.

Since we were examining paired samples of tumor and normal mucosal tissue from the same patients, we also performed a test of the seven adenocarcinoma cases using paired statistics in edgeR (Figure 2B). A total of 118 miRs were identified as significantly dysregulated under the same conditions as for the non-paired analysis (Table S3). Of these, there were 81 miRs that were not identified in the non-paired analysis, and a common overlap of 37 for both approaches. This confirms the prediction that the non-paired analysis is the more conservative test, although there are 15 miRs identified as dysregulated in the DESeq non-paired test which were not identified by the paired analysis in edgeR (Figure 3).

It is apparent that there are 37 common miRs found to be significantly dysregulated when using both statistical approaches (Table 2). There is approximately equal distribution between the down- and up-regulated miRs. There are both lowly (approximately 10–10 000 absolute reads) and highly (approximately 10 000–5 000 000 absolute reads) expressed miRs represented in the common miR subset, two notable examples being miR-7 and miR-1, respectively. When looking at expression levels globally in terms of all identified miRs, there is a global up-regulation of expression in the tumor compared to that of normal mucosa (considered from the paired analysis of the adenocarcinomas).

**Table 2:** Intersect of significant miRs from the adenocarcinoma cases when using non-paired (DESeq) and paired (edgeR) analysis approach.

miRNA	Log2FC	FDR	NET	Selected relevant cancers with references
**Down-regulated**				
hsa-miR-1	−2,0	9,0E-03	No	**Down-regulated** in colorectal [17] and other cancers [32], [33].
hsa-miR-139-5p	−2,7	5,6E-04	No	**Down-regulated** in gastric [31] and other cancers [32], [59].
hsa-miR-145	−1,7	2,6E-02	No	**Down-regulated** in colorectal [16], [34], [36] and other cancers.
hsa-miR-195	−2,3	2,2E-03	No	**Down-regulated** in colorectal cancer [39], [40].
hsa-miR-363	−1,9	2,9E-02	No	**Down-regulated** in colorectal cancer [17].
hsa-miR-378	−1,7	3,6E-02	No	**Down-regulated** in colorectal cancer [41].
hsa-miR-378c	−1,9	2,3E-02	No	**Down-regulated** in colon cancer [41] and gastric cancer [31].
hsa-miR-383	−1,7	7,3E-02	No	**Down-regulated** in gastric cancer [45].
hsa-miR-422a	−2,4	2,2E-03	No	**Down-regulated** in colon cancer [60].
hsa-miR-486-5p	−2,1	4,7E-02	No	**Down-regulated** in colon and other cancers [61].
hsa-miR-490-3p	−1,8	6,5E-02	No	Few if any references.
hsa-miR-551b	−3,7	4,4E-04	No	**Down-regulated** in colon cancer [17].
hsa-miR-628-3p	−6,2	4,1E-04	No	Few if any references.
hsa-miR-628-5p	−1,7	4,0E-02	No	Few if any references.
hsa-miR-1297	−6,8	2,9E-02	No	Few if any references.
hsa-miR-3151	−3,1	1,6E-02	No	Few if any references.
hsa-miR-3163	−2,1	4,9E-02	No	Few if any references.
hsa-miR-3622a-5p	−2,0	2,8E-02	No	Few if any references.
hsa-miR-3656	−2,3	1,9E-02	No	Few if any references.
**Up-regulated**				
hsa-miR-7	3,5	6,9E-07	Yes	**Up- and down-regulated** in different cancers (see text).
hsa-miR-96	3,2	1,9E-06	Yes	**Up-regulated** in colon cancer [14], [17], [50].
hsa-miR-105	4,0	7,5E-02	No	Few if any references.
hsa-miR-135b	4,2	2,1E-08	No	**Up-regulated** in colon cancer [14], [17], [41], [50].
hsa-miR-296-3p	1,9	3,5E-02	No	**Up-regulated** in immortalized human cells [52].
hsa-miR-483-3p	3,6	5,1E-05	No	**Up-regulated** in colon, pancreas and other cancers [62], [63].
hsa-miR-493	3,4	4,2E-06	No	**Up-regulated** in colon cancer [50].
hsa-miR-549	5,8	8,6E-06	No	Few if any references.
hsa-miR-552	4,3	1,7E-07	No	**Up-regulated** in MMR proficient colon cancers and **down-regulated** in MMR deficient colon cancers [17].
hsa-miR-584	3,4	1,9E-06	No	**Up-regulated** in colon cancer [17].
hsa-miR-592	3,8	7,0E-06	No	**Up-regulated** in MMR proficient colon cancers and **down-regulated** in MMR deficient colon cancers [17].
hsa-miR-1247	1,9	5,3E-02	No	Methylated gene (low expression) in HCT116 cells [57]
hsa-miR-1269	4,4	6,3E-07	Yes	Few if any references.
hsa-miR-1827	3,0	3,3E-04	Yes	Few if any references.
hsa-miR-3144-3p	2,8	4,8E-02	No	Few if any references.
hsa-miR-3177	3,2	1,4E-02	Yes	Few if any references.
hsa-miR-3180-3p	2,6	4,8E-02	No	Few if any references.
hsa-miR-4326	2,5	8,4E-02	No	Few if any references.

Adjusted for multiple testing using Benjamini and Hochberg, false discovery rate (FDR) < 0.1. Logarithmic fold change (FC) relative to normal mucosa and FDR from paired analysis using edgeR. miRs also significant in the analysis of the neuroendocrine tumor (NET) is indicated.

The high-throughput sequencing was experimentally validated using a quantitative polymerase chain reaction for selected miRs and tissue specimens (Figure S1). Our results are in line with previous inter-platform validation results [28]: the results between the different methods correlate, but this correlation is far from perfect.

## Discussion

Several studies have found that miRs are globally down-regulated in different cancers, with a correlation between the degree of differentiation and global expression levels of miRs. Although it has been indicated that global down-regulation promotes cell transformation and tumorigenesis [10], [15], [29], a large expression profiling study of solid tumors by Volinia *et al*. did not observe down-regulation of miRs as previously reported [30]. Our study suggests that global down-regulation is not the case for the colorectal adenocarcinomas in our cohort, even though a substantial number of individual miRs are down-regulated in the adenocarcinomas relative to the normal samples.

According to the miRecords database [31], miR-1 has 117 validated targets and could potentially interact with several important genes in carcinogenesis of colorectal cancer. In a study from 2009, miR-1 and miR-551b (among others) were found to have lower expression in embryonic stem cells relative to differentiated cells and in colorectal cancer relative to normal mucosa [17]. This is consistent with our findings of down-regulated miR-1 and miR-551b in the colorectal adenocarcinomas. Down-regulated miR-1 is also observed in the neuroendocrine case. miR-1 has further been reported to be down-regulated and suggested a tumor-suppressive function by targeting the transgelin 2 gene (*TAGLN2*) in bladder cancer [32] and head and neck squamous cell carcinomas [33].

miR-145 is down-regulated in the adenocarcenomas of our study, and this miR has frequently been associated with down-regulation in colorectal cancers [16], [34], [35], [36]. It is thought to have a tumor-suppressor role, partly by targeting the insulin receptor substrate 1 (ISR-1) and type I insulin-like growth factor receptor (IGF-IR). Loss of miR-145 inhibition increases anti-apoptotic signals in the cell and promote cell growth [37], [38].

In a study from 2010, miR-195 was found to be down-regulated in 81 colorectal cancer tissues compared to matched normal mucosa and this is in accordance with our results for the adenocarcinomas. This miR is believed to target Bcl-2 and hence exert its pro-apoptotic function when physiologically regulated [39]. Another study showed that reduced expression of miR-195 occurred more often in patients with lymph node metastasis and advanced tumor stage. Low expression levels were also poor predictors of overall survival [40].

In two minor studies, one of colon cancer without lymph node metastasis [41] and the other of gastric cancer [31], miR-378 was found to be down-regulated in the tumors compared to normal adjacent tissue as seen in our study. It has however been reported that miR-378 promotes cell survival and tumor growth by targeting Sufu and Fus-1 [42] and it may also play a modifying role with other miRs in angiogenesis [43]. There is further evidence that the Myc/miR-378/TOB2/cyclin D1 functional module regulates oncogenic transformation [44].

miR-383 is also down-regulated in the adenocarcinomas compared to normal tissue. To our knowledge this has not been reported for colorectal adenocarcinomas, but has been observed in a small study on gastric cancer [45]. There is good concordance between our findings of down-regulated miRs in colorectal adenocarcinomas and previously published reports. As well as the miRs described above, we identified a significant number of other uniformly down-regulated miRs, less referred to in the literature; -139-5p, -363, -422a, -486-5p, -490-3p, -628-3p, -628-5p, -1297, -3151, -3163, -3622a-5p and -3656 (Table 2). This highlights the potential of high throughput sequencing as a tool for identifying miRs potentially related to carcinogenesis that could have been missed using array based technology.

miR-7 has a functional role in the differentiation of epithelial cells in the intestine, reviewed by Tazawa *et al*. [46]. It is thought to regulate the expression of transmembrane glycoprotein CD98 which has an important role in cell adhesion through interaction with integrin beta-1. Up-regulation of miR-7 suppresses CD98 expression in Caco2-BBE cells and hence modulates beta-1-integrin-laminin-1 interactions. This may further affect proliferation and differentiation of enterocytes during migration across the crypt-villus axis [47]. miR-7 has been reported to function as a tumor-suppressor in schwannomas [48] but as an oncogene in lung squamous cell carcinomas [49]. There is emerging evidence that increased EGFR expression is associated with an increased miR-7 level, at least in squamous cell carcinomas. The miR-7 in turn targets Ets2 repressor factor (ERF), attenuates EGFR expression and modulates cell growth [49]. It is therefore possible that miR-7 may function in several feedback and feedforward loops, both as tumor-suppressor and oncogene depending on tumor type. Our findings strongly suggest that miR-7 is up-regulated in both colorectal adenocarcinomas and in the neuroendocrine case. Based on previous findings and published validated targets for miR-7 such as *EGFR*, *PAK1*, *RAF1, IRS1/2* and *CD98* [31], it is fair to hypothesize that this miR may be involved in regulating intracellular signaling, growth and differentiation of colorectal cancers.

The expressions of miR-96, miR-135b and miR-493 were increased in several studies on colorectal cancer, as well as in our study [14], [17], [50]. miR-135 has been shown to directly target the 3′ UTR of *APC* and induce the downstream Wnt pathway [51]. Our results also show that miR-552 and -592 expressions were increased in the adenocarcinomas compared to normal tissues. Previously published data for these two miRs demonstrated an up-regulation in colorectal cancers with proficient mismatch repair status (MMR) but down-regulation in MMR deficient tumors relative to normal colon tissue [17]. Yoon *et al* observed that miR-296 interacted with the 3′ UTR of the *CDKN1A* (p21/WAF1) gene, and that this miR was frequently up-regulated during immortalization of human cells [52]. Interestingly, we also observe an up-regulation of miR-296-3p. This miR could as such contribute to carcinogenesis by inhibiting the p53-p21/WAF1 pathway.

There are not many publications on the function of miR-549 (Chr15 in *KIAA1199*), and to our knowledge none in relation to colorectal cancer. Interestingly, the gene transcribing this miR is localized in the *KIAA1199* gene. This gene of uncertain function has previously been reported to be strongly up-regulated in colorectal adenomas (n = 32) and carcinomas (n = 25) analyzed in a study by Sabates-Bellver *et al*. The study also show that the expression of 19 Wnt targets was closely correlated with up-regulation of *KIAA1199*, and that the expression in normal mucosa was limited to cells in the lower portion of colonic crypts [53], [54]. The over-expression of *KIAA1199* has later been confirmed for colonic adenomas [55] and gastric cancer [56]. If *KIAA1199* and miR-549 are co-transcribed, this may explain the increased expression levels of miR-549 found in our study. Furthermore, as the up-regulation seems to be an early event from previously published studies, the miR-549 could potentially be a surrogate biomarker for adenoma development and early adenocarcinoma stages. This should be further investigated in larger studies.

A study on epigenetically silenced miRs in colorectal cancer found that miR-1247 was methylated in HCT116 cells. HCT116 and DLD1 cells were then transfected with a miR-1247 mimic which resulted in a significant decrease in cell growth and metabolic activity in both cell lines. DKO cells (HCT 116 cells deleted for DNA methyltransferase) did however not decrease cell growth when introduced to the mimic, but caused impaired cell migration [57]. The role of this miR still remains unclear, but it has been hypothesized to function as a tumor suppressor. We found this miR to be up-regulated in the adenocarcinomas, which could indicate different targets in the pure cell lines compared to that of an organized tumor tissue.

Finally there are few, if any, reports on the function and role in colonic adenocarcinomas of the following miRs up-regulated in our study: -105, -483-3p, -584, -1269, -1827, -3144-3p, -3177, -3180-3p and -4326 (Table 2).

As we included a neuroendocrine tumor (NET) in this study, we could take advantage of analyzing this separately using a similar statistical approach as for the adenocarcinomas. Although, we are working partially without replicates, the DESeq tool can handle this challenge [25]. NETs are rare tumors that originate from neuroendocrine cells at different sites in the body, including the gastrointestinal site. There is an increasing incidence, partly due to better registration and possibly better diagnostic tools [58]. However, very few studies have examined the miR expression in NET. In our study, the NET shares a few significant miRs with the adenocarcinomas, but what is more striking are some of the unique and highly expressed miRs (Table S2). These have large fold changes compared to non-paired normal tissues and also a higher relative expression compared to the adenocarcinomas. The expression pattern of miRs in the NET differs extensively from the normal mucosa. This may of course be partly due to the neuroendocrine tissue itself which is functionally and genetically different from normal epithelium and stroma. Nevertheless, the identified miRs may potentially help differentiate between malignant neuroendocrine cells of the colon and normal mucosa (as our data suggests), and possibly also between benign neuroendocrine cells and normal mucosa (no data). The sample size of one means that the NET data can only be considered indicative. However, in our opinion, the substantial differences in the sets of differentially regulated miRs between the two types of cancers deserve to be reported. Our observation suggests that it may be fruitful to further investigate these miR markers as they may be useful in establishing the origin of poorly differentiated colorectal cancers.

Microdissection of tumor tissue has not been the standard in studies previously performed. We have however examined the histopathology of the tissue specimens, and estimated the tumor and stromal percentages. The tumor percentage was about 67% in average, well above the average for a subgroup of the KAM cohort (n = 139) which was 49% +/– 24% (data not published). Unfortunately, one sample in the dataset was aberrant with a low tumor percentage (Table 1), and this is a weakness of our study. Ideally, the study samples should have had a more homogenous tumor population. There is however a notion that the normal mucosa mainly consists of epithelial cells and stroma. When comparing the tumor tissue and normal mucosa, we are mainly comparing tumor cells (with varying amounts of stroma) with epithelial cells and stroma in the normal mucosa. As such, we believe the effect of a too low tumor percentage will be false negative results.

In high-throughput experiments (whether array or sequencing based), it is common to perform a validation experiment using another technology. We performed such a validation experiment using a quantitative polymerase chain reaction for selected miRs and tissue specimens (Figure S1). The results show a positive correlation between the two different technology platforms. There are seven miRs for which the fold changes are very different in the validation. Such differences in fold change between technology platforms are not unusual as demonstrated by a study of differential miR expression using the Affymetrix, Agilent, and Illumina microarray platforms, as well as quantitative PCR and high–throughput sequencing [28]. Although of concern, this observation does not invalidate the results obtained. Indeed, it has been observed that methods for miR gene expression profiling are strongly biased toward certain miRs, preventing the accurate determination of absolute numbers. The observed bias is strongly determined by the method used for library preparation. However, since the biases are systematic and highly reproducible for a given technology, gene expression profiling is suited for determining relative expression differences between samples as long as the same technology is used across samples [23]. In our study, due to the large amounts of cDNA required for the high-throughput sequencing analysis, we did not have sufficient cDNA available for quantitative PCR validation for all patients. We therefore had to do a second round of RNA extraction from adjacent tissue where available. Any heterogeneity between the adjacent tissues may add to the variability observed in the validation data (Figure S1).

This study is to our knowledge unique in that global high-throughput sequencing has been used to characterize miR expression in paired colorectal cancer tissue and adjacent normal mucosa. Although preliminary, we believe that the results may serve as a robust training set for a larger cohort study. We utilized paired and non-paired statistics, and identified 37 miRs that are dysregulated in the seven adenocarcinoma cases in both statistical approaches; 19 down-regulated and 18 up-regulated. Our comprehensive survey of differentially expressed miRs confirms some existing findings. We have also discovered 16 dysregulated miRs which, to our knowledge, have not previously been associated with colorectal carcinogenesis. Our results indicate that these may be important regulators and that further investigations into potential miR targets and their possible use as predictive or prognostic markers are warranted. Particularly interesting is the miR-549 gene located in *KIAA1199* which itself has previously been associated with up-regulation in colonic adenomas and carcinomas. If the miR is co-transcribed, it could be a potential surrogate marker for early disease detection in body fluids or feces. The study has also shed new light on potential miR biomarkers that seem to be specific for NETs in the colon.

## Materials and Methods

### Cohort

Eight colorectal cancer patients were selected from a Norwegian colorectal cancer cohort (*Kolorectalcancer, arv og miljø,* KAM) based on the parameters age and gender. All patients were male with an average age of 60 years. All of the tissue samples were extracted from surgical specimens. The normal mucosa was collected in a distal part of the bowel close to the resection margins. Samples were subsequently frozen in liquid nitrogen and stored in a freezer at –80 degrees Celsius. Seven of the patients were confirmed to have adenocarcinomas and one was characterized as a neuroendocrine tumor by histopathological examination. Clinical and histopathological characteristics of the patients are summarized in Table 1.

### RNA Extraction and Digital Sequencing

Total RNA from the patients was extracted from 10 frozen sections of 10 µm for tumor and normal tissue respectively using the mirVana kit (Ambion, TX, USA) according to the manufacturer’s protocol. Some samples were concentrated in a vacuum centrifuge to obtain the necessary concentration of 1 µg/µl. The presence of small RNA was confirmed on a Bioanalyzer 2100 (Agilent, CA, USA) without sign of degradation when evaluating OD ratio 260/280. The starting amount was 10 µg of total RNA, and the preparation protocol was performed according to the manufacturer’s recommendations. Small RNA was isolated from total RNA on a 15% Novex TBE-Urea PAGE gel. The area representing band size of 18–30 nucleotides (nt) was cut out and fragmented, RNA was eluted in 0.3 M NaCl and purified on a Spin X column. The 5′-adapter was ligated for 6 hours at 20°C. Small RNA with ligated 5′-adapter was isolated on a 15% Novex TBE-Urea PAGE gel (Invitrogen, CA, USA). The 40–60 nt band was cut out and fragmented, RNA was eluted in 0.3 M NaCl and purified on a Spin X column. The 3′-adapter was ligated for 6 hours at 20°C. Small RNAs with ligated 5′- and 3′-adapters were isolated on a 10% Novex TBE-Urea PAGE gel, the 70–90 nt band was cut out and fragmented, RNA was eluted in 0.3 M NaCl and cleaned on a Spin X column. Then GlycoBlue and ethanol were added followed by precipitation for 30 minutes at –80°C and centrifugation at 14 000 rpm for 25 minutes. The RNA pellet was dissolved in 4.5 µl RNase free water. Reverse transcription and amplification was carried out and the cDNA was separated on a 6% Novex TBE PAGE gel. The amplified cDNA band was cut out and fragmented; RNA was eluted in Gel Elution Buffer and purified on a Spin X column. Then glycogen and ethanol were added for precipitation followed by centrifugation at 14 000 rpm and 4°C for 20 minutes. The cDNA pellet was dissolved in 10 µl Resuspension Buffer. The cDNA library generated was evaluated with a quantitative real-time PCR to ensure acceptable quality and confirm that adapters were correctly added. The high-throughput sequencing of the cDNA was done in a 36 bp single read run on an Illumina Genome Analyzer IIx (Illumina, CA, USA). Image analysis and base calling was performed with the Illumina GA pipeline software version 1.5.1. Sequences with a chastity less than 0.6 on two or more bases among the first 25 bases were filtered out (this is the default setting for the software).

### Experimental Validation with RT Real-time PCR

A total of six miRs (miR-1, -21, -143, -145, -423-5p and -192) were selected for experimental validation using a reverse transcription (RT) real-time PCR protocol. Total RNA from three patients (six tissue specimens) was re-extracted as previously described due to shortage of total RNA from first extraction batch. cDNA was constructed from total RNA using the TaqMan MicroRNA Reverse Transcription Kit and Megaplex RT Primers Pool A (Applied Biosystems). Pre-amplification of cDNA was performed using Megaplex PreAmp Primers (Applied Biosystems) to increase the starting amount prior to gene expression analysis. It enables an unbiased pre-amplification prior to loading the TaqMan MicroRNA Array according to the manufacturer’s instructions. Single sequence-specific miR real-time PCR assays were used to quantitate each individual mature miRNA (Applied Biosystems, Assay IDs; 002222, 000397, 002249, 002278, 002340 and 000491) using a TaqMan MGB probe. Expression of RNU44 and RNU48 were tested across a set of miR samples (n = 20) from colorectal cancer patients, and they were both found to have stable expression across samples. RNU48 was used as endogenous control. The ΔΔCt method was used for calculating the relative expression of a given miR between a paired normal and tumor sample. Fold change was further calculated as 2^-ΔΔC^. For the digital gene expression data, the count data was normalized to the estimated size factors (DESeq). Fold change was calculated as the ratio between normalized count data for tumor and normal samples. Fold changes for the high-throughput sequencing and quantitative PCR were log transformed and plotted with an expected trend line (Figure S1).

### Data Analysis

Data from the high throughput sequencing was obtained in FASTQ format, one data file per sequencing lane (n = 16). The sequencing adaptors were subsequently clipped and removed using the FASTX-Toolkit (http://hannonlab.cshl.edu/fastx_toolkit/), allowing no mismatches for adaptor identification. The remaining sequencing data was further collapsed and counted into groups of identical sequences. The sequencing data was further processed using the miRanalyzer tool version 0.2 [24]. This tool allows for the identification of validated miRs from the miRBase (release 16) data repository [8] and includes a machine learning algorithm for the prediction of novel miRs. It also evaluates sequence alignment to other entities through the databases RefSeq and Rfam. Sequence data was aligned to the Homo Sapiens hg18 genome reference allowing for one mismatch.

Differential expression (DE) of identified miRs from miRBase was calculated with R version 2.13.0 using DESeq version 1.4.1 [25] and edgeR version 2.2.5 [26] available in Bioconductor version 2.8. Both tools utilize a negative binomial distribution for modeling read counts per miR and implement a method for normalizing the counts. We began by ignoring the pairing information between the samples: differential expression (fold change) of known miRs was analyzed between the group of adenocarcinoma (n = 7) and normal mucosa (n = 8), subsequently between the neuroendocrine case (n = 1) and normal mucosa (n = 8) using DESeq. A diagnostic plot provided in the supplementary materials for the fit of the variance function (Figure S2) shows how the use of the negative binomial model enables a good estimation of the variance (something that would not have been possible with a Poisson model). P-values are adjusted for multiple testing using the Benjamini and Hochberg method [27]. Only miRs with a fold change with adjusted P-value with false discovery rate (FDR) < 0.1 are considered significant [25]. Since all samples of cancerous and normal mucosal tissues are paired from the same patients, we also performed a test of all adenocarcinoma cases using paired statistics in edgeR with a generalized linear model (GLM) method. This method was adjusted for multiple testing as above. The miR count data for all samples (Dataset S1) and the R code (Text S1) are available online.

### Ethics Statement

We obtained written informed consent from all the participants involved in the study. This project has been approved by Regional komite for medisinsk og helsefaglig forskningsetikk Sør-Øst (The Ethics Committee REK Sor-Ost A). Review board: Professor G. Nicolaysen (Leader of Ethics Committee), J. Hardang (Senior Consultant) and K. Ore (Consultant). Ref.: 2009/2021/S-98198.

## Supporting Information

Figure S1 Experimental validation of selected miRs and cases. Plot of log transformed fold change from quantitative polymerase chain reaction (qPCR) versus high-throughput sequencing (HTS). Expected trend line included.
(PDF)

Figure S2 Diagnostic plot produced in DESeq illustrating the fit of the variance function (base variance versus base levels). The red line shows the fit from the local regression. Black dotted line shows mean = variance which is the expected fit for Poisson distributed data.
(PDF)

Table S1 Results from the DESeq differential expression analysis of the adenocarcinoma cases.
(PDF)

Table S2 Results from the DESeq differential expression analysis of the neuroendocrine case.
(PDF)

Table S3 Results from the edgeR differential expression analysis of the adenocarcinoma cases.
(PDF)

Dataset S1 miR count data for all samples in the study. Output from processed sequencing data aligned to the Homo Sapiens hg18 genome reference using the miRanalyzer tool version 0.2.
(ZIP)

Text S1 R code for calculating differential expression.
(PDF)
