# Supplementary figures and images for: Differential Expression of miRNAs in Colorectal Cancer: Comparison of Paired Tumor Tissue and Adjacent Normal Mucosa Using High-Throughput Sequencing
Source: PLoS One. 2012 Apr 17;7(4):e34150. doi: 10.1371/journal.pone.0034150 (PMC3328481; doi:10.1371/journal.pone.0034150)

# HTS vs qPCR

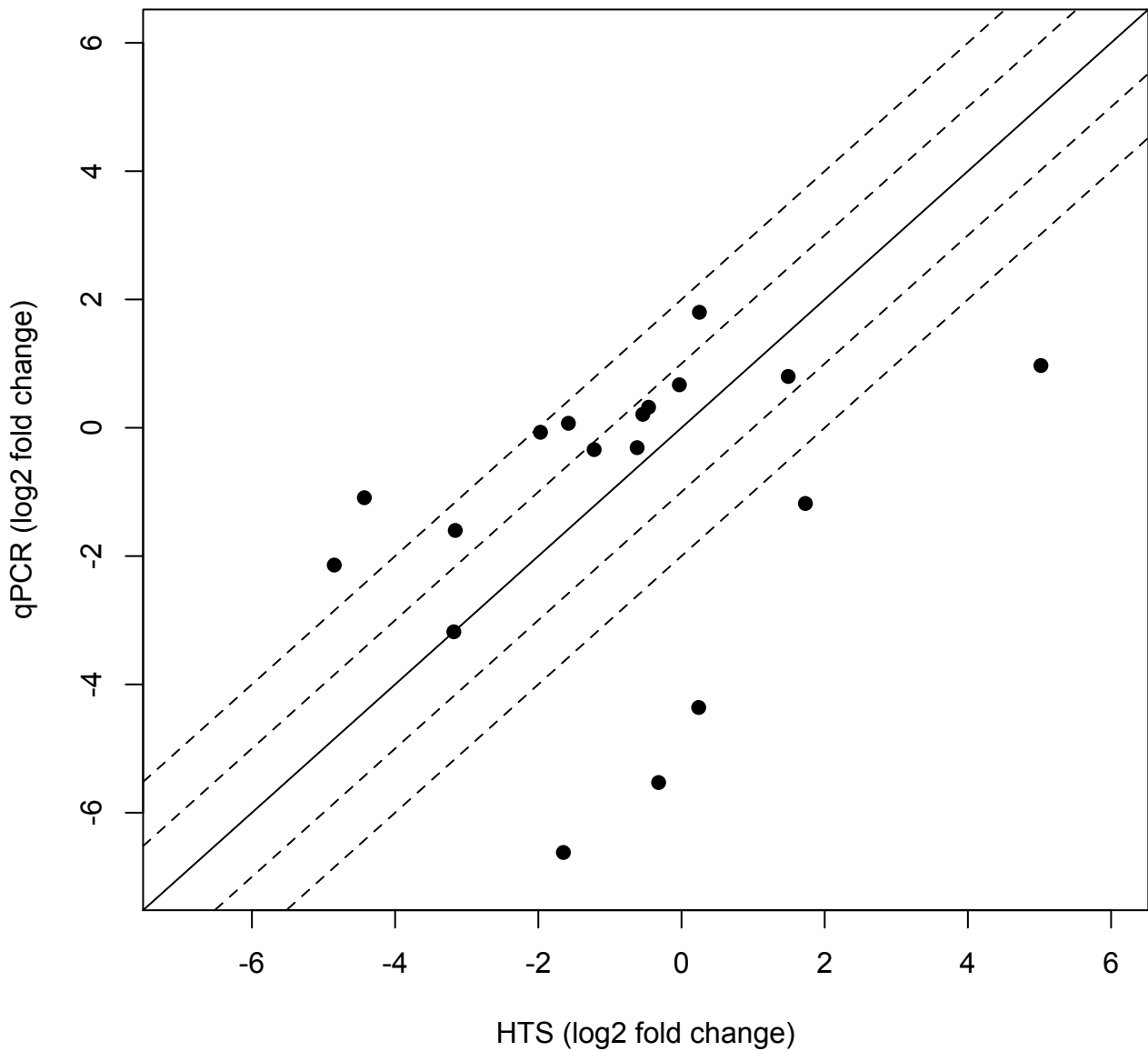

Supplement: Figure S1 — Experimental validation of selected miRs and cases. Plot of log transformed fold change from quantitative polymerase chain reaction (qPCR) versus high-throughput sequencing (HTS). Expected trend line included. (PDF) [file pone.0034150.s001.pdf]

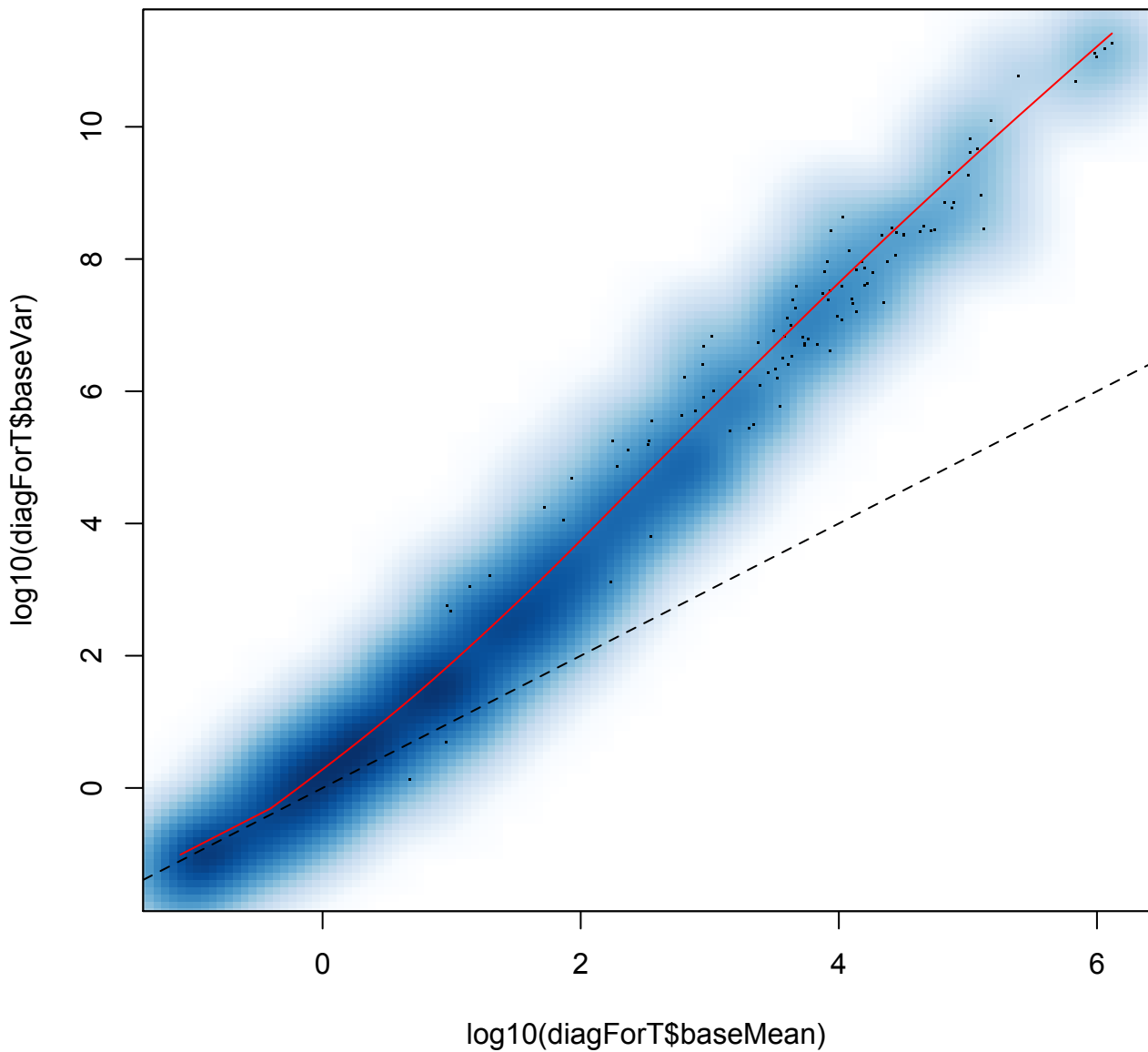

Supplement: Figure S2 — Diagnostic plot produced in DESeq illustrating the fit of the variance function (base variance versus base levels). The red line shows the fit from the local regression. Black dotted line shows mean = variance which is the expected fit for Poisson distributed data. (PDF) [file pone.0034150.s002.pdf]
