# Supplementary material for: Differential Expression of miRNAs in Colorectal Cancer: Comparison of Paired Tumor Tissue and Adjacent Normal Mucosa Using High-Throughput Sequencing
Source: PLoS One. 2012 Apr 17;7(4):e34150. doi: 10.1371/journal.pone.0034150 (PMC3328481; doi:10.1371/journal.pone.0034150)
Supplement: Table S1 — Results from the DESeq differential expression analysis of the adenocarcinoma cases. (PDF) [file pone.0034150.s003.pdf]

Supporting Information: Table S1

miRNA	Log2 Fold Change	FDR
hsa-miR-628-3p	-7,21	7,01E-09
hsa-miR-483-3p	4,59	7,41E-09
hsa-miR-206	4,10	3,84E-08
hsa-miR-1269	4,33	9,81E-08
hsa-miR-187	-3,97	2,14E-07
hsa-miR-215	-3,97	2,15E-07
hsa-miR-1	-2,13	2,70E-07
hsa-miR-204	4,20	5,50E-07
hsa-miR-135b	3,74	1,07E-05
hsa-miR-3180-3p	3,57	1,91E-05
hsa-miR-552	3,24	6,15E-05
hsa-miR-7	3,33	6,37E-05
hsa-miR-551b	-4,07	1,20E-04
hsa-miR-767-5p	3,36	1,48E-04
hsa-miR-1275	-3,52	2,11E-04
hsa-miR-184	-2,83	3,45E-04
hsa-miR-3656	-3,61	5,59E-04
hsa-miR-96	2,87	1,52E-03
hsa-miR-105	3,65	1,81E-03
hsa-miR-549	5,73	1,84E-03
hsa-miR-422a	-3,02	2,32E-03
hsa-miR-584	2,65	2,52E-03
hsa-miR-592	3,08	2,63E-03
hsa-miR-378	-2,14	2,98E-03
hsa-miR-378c	-2,55	4,38E-03
hsa-miR-490-3p	-2,30	4,83E-03
hsa-miR-139-5p	-2,63	5,16E-03
hsa-miR-1297	-Inf	6,13E-03
hsa-miR-3622a-5p	-2,62	6,38E-03
hsa-miR-1827	3,45	9,36E-03
hsa-miR-3151	-3,77	9,36E-03
hsa-miR-383	-2,21	9,86E-03
hsa-miR-145	-2,19	1,05E-02
hsa-miR-301b	2,87	1,08E-02
hsa-miR-195	-2,20	1,21E-02
hsa-miR-3180-5p	2,69	1,21E-02
hsa-miR-363	-2,17	1,23E-02
hsa-miR-24	-1,18	1,63E-02
hsa-let-7d	-1,54	2,02E-02
hsa-miR-628-5p	-1,94	2,33E-02
hsa-miR-1247	2,42	2,57E-02
hsa-miR-493	2,57	2,73E-02
hsa-miR-296-3p	2,18	4,62E-02
hsa-miR-3914	-2,51	4,66E-02
hsa-miR-214	-1,54	4,78E-02
hsa-miR-4326	2,81	5,57E-02
hsa-miR-483-5p	1,55	5,73E-02
hsa-miR-486-5p	-2,22	9,38E-02
hsa-miR-3936	-1,90	9,49E-02
hsa-miR-3177	2,50	9,49E-02
hsa-miR-3144-3p	2,58	9,49E-02
hsa-miR-3163	-2,85	9,65E-02
