# Supplementary material for: Differential Expression of miRNAs in Colorectal Cancer: Comparison of Paired Tumor Tissue and Adjacent Normal Mucosa Using High-Throughput Sequencing
Source: PLoS One. 2012 Apr 17;7(4):e34150. doi: 10.1371/journal.pone.0034150 (PMC3328481; doi:10.1371/journal.pone.0034150)
Supplement: Table S2 — Results from the DESeq differential expression analysis of the neuroendocrine case. (PDF) [file pone.0034150.s004.pdf]

Supporting Information: Table S2

miRNA	Log2 Fold Change	FDR
hsa-miR-653	11,27	2,70E-20
hsa-miR-7	9,01	4,11E-18
hsa-miR-489	8,65	2,92E-15
hsa-miR-1224-5p	7,11	1,31E-12
hsa-miR-1269	6,98	2,54E-12
hsa-miR-137	7,08	4,67E-12
hsa-miR-375	6,08	8,51E-12
hsa-miR-615-3p	7,60	3,75E-11
hsa-miR-153	6,19	2,24E-08
hsa-miR-3065-3p	5,79	2,67E-07
hsa-miR-204	5,54	3,83E-07
hsa-miR-129-3p	5,23	5,21E-07
hsa-miR-615-5p	5,46	5,21E-07
hsa-miR-330-5p	5,42	2,27E-06
hsa-miR-1468	5,11	2,53E-06
hsa-miR-129-5p	4,61	1,31E-05
hsa-miR-1827	4,04	1,67E-04
hsa-miR-3065-5p	4,58	2,19E-04
hsa-miR-196a	3,84	4,66E-04
hsa-miR-96	3,96	5,06E-04
hsa-miR-1179	3,97	2,04E-03
hsa-miR-1224-3p	6,41	3,03E-03
hsa-miR-1301	3,48	5,60E-03
hsa-miR-135a	3,54	8,12E-03
hsa-miR-190b	3,82	9,05E-03
hsa-miR-95	3,12	1,19E-02
hsa-miR-744	3,10	1,63E-02
hsa-miR-3200-3p	3,56	2,43E-02
hsa-miR-3177	4,27	2,67E-02
hsa-miR-889	3,02	4,37E-02
hsa-miR-1180	3,09	5,15E-02
hsa-miR-668	4,34	5,67E-02
hsa-miR-885-3p	3,03	5,67E-02
hsa-miR-182	2,64	5,88E-02
hsa-miR-330-3p	2,78	6,12E-02
hsa-miR-488	Inf	6,39E-02
hsa-miR-3131	2,60	6,58E-02
hsa-miR-651	2,92	9,24E-02
