# Supplementary material for: Differential Expression of miRNAs in Colorectal Cancer: Comparison of Paired Tumor Tissue and Adjacent Normal Mucosa Using High-Throughput Sequencing
Source: PLoS One. 2012 Apr 17;7(4):e34150. doi: 10.1371/journal.pone.0034150 (PMC3328481; doi:10.1371/journal.pone.0034150)
Supplement: Table S3 — Results from the edgeR differential expression analysis of the adenocarcinoma cases. (PDF) [file pone.0034150.s005.pdf]

Supporting Information: Table S3

miRNA	Log2 Fold Change	FDR
hsa-miR-135b	4,24	2,09E-008
hsa-miR-552	4,30	1,71E-007
hsa-miR-1269	4,37	6,34E-007
hsa-miR-7	3,50	6,93E-007
hsa-miR-584	3,39	1,87E-006
hsa-miR-96	3,22	1,87E-006
hsa-miR-493	3,45	4,17E-006
hsa-miR-592	3,77	7,04E-006
hsa-miR-549	5,81	8,61E-006
hsa-miR-483-3p	3,56	5,11E-005
hsa-miR-1827	3,00	3,33E-004
hsa-miR-628-3p	-6,17	4,07E-004
hsa-miR-551b	-3,65	4,37E-004
hsa-miR-139-5p	-2,66	5,56E-004
hsa-miR-542-3p	2,28	1,49E-003
hsa-miR-224	2,35	1,69E-003
hsa-miR-203	2,69	2,16E-003
hsa-miR-889	2,27	2,16E-003
hsa-miR-195	-2,31	2,16E-003
hsa-miR-422a	-2,38	2,16E-003
hsa-miR-134	2,09	3,32E-003
hsa-miR-31	2,21	3,34E-003
hsa-miR-146b-5p	2,06	4,86E-003
hsa-miR-1295	3,21	5,12E-003
hsa-miR-124	-2,32	7,12E-003
hsa-miR-146a	2,19	8,23E-003
hsa-miR-651	2,31	8,47E-003
hsa-miR-1	-2,04	8,98E-003
hsa-miR-95	1,98	9,47E-003
hsa-miR-146b-3p	1,96	1,08E-002
hsa-miR-3177	3,18	1,37E-002
hsa-miR-3151	-3,11	1,63E-002
hsa-miR-496	2,17	1,63E-002
hsa-let-7i	1,80	1,72E-002
hsa-miR-200a	2,19	1,72E-002
hsa-miR-3168	1,88	1,84E-002
hsa-miR-382	1,77	1,87E-002
hsa-miR-3656	-2,29	1,92E-002
hsa-miR-675	2,25	2,09E-002
hsa-miR-19a	2,01	2,26E-002
hsa-miR-378c	-1,86	2,26E-002
hsa-miR-450a	1,81	2,33E-002
hsa-miR-155	1,79	2,41E-002
hsa-miR-3117	1,97	2,61E-002
hsa-miR-145	-1,72	2,63E-002
hsa-miR-429	2,07	2,83E-002
hsa-miR-3622a-5p	-1,99	2,85E-002
hsa-miR-363	-1,86	2,91E-002
hsa-miR-1297	-6,81	2,91E-002
hsa-miR-3162	2,54	2,93E-002
hsa-miR-409-5p	1,71	3,18E-002

Supporting Information: Table S3

hsa-miR-3662	4,27	3,30E-002
hsa-miR-1246	1,81	3,37E-002
hsa-miR-296-3p	1,86	3,51E-002
hsa-miR-589	1,67	3,61E-002
hsa-miR-127-5p	1,66	3,63E-002
hsa-miR-378	-1,69	3,63E-002
hsa-miR-508-3p	2,42	3,72E-002
hsa-miR-762	6,99	3,92E-002
hsa-miR-628-5p	-1,66	3,99E-002
hsa-miR-194	1,86	4,04E-002
hsa-miR-486-5p	-2,12	4,69E-002
hsa-miR-769-5p	1,58	4,77E-002
hsa-miR-200b	1,97	4,77E-002
hsa-miR-3180-3p	2,60	4,77E-002
hsa-miR-345	1,57	4,77E-002
hsa-miR-92a	1,56	4,77E-002
hsa-miR-3144-3p	2,82	4,77E-002
hsa-miR-3163	-2,14	4,89E-002
hsa-miR-654-5p	1,80	4,89E-002
hsa-miR-21	1,49	4,97E-002
hsa-miR-449a	2,35	5,00E-002
hsa-miR-1247	1,89	5,28E-002
hsa-miR-20b	-1,86	5,76E-002
hsa-miR-330-5p	1,72	6,03E-002
hsa-miR-34a	1,49	6,42E-002
hsa-miR-3652	2,03	6,47E-002
hsa-miR-3620	3,69	6,47E-002
hsa-miR-490-3p	-1,79	6,54E-002
hsa-miR-144	-2,02	6,54E-002
hsa-miR-1301	1,47	6,72E-002
hsa-miR-744	1,40	6,80E-002
hsa-miR-370	1,55	6,84E-002
hsa-miR-423-5p	1,48	6,96E-002
hsa-miR-1254	1,64	6,96E-002
hsa-miR-1292	1,67	7,14E-002
hsa-miR-409-3p	1,45	7,29E-002
hsa-miR-19b	1,66	7,29E-002
hsa-miR-383	-1,67	7,29E-002
hsa-miR-105	3,97	7,49E-002
hsa-miR-653	2,81	7,49E-002
hsa-miR-720	1,51	7,50E-002
hsa-miR-548o	1,69	7,50E-002
hsa-miR-622	6,54	7,55E-002
hsa-miR-3171	6,28	7,58E-002
hsa-miR-129-3p	-1,55	7,65E-002
hsa-miR-29c	-1,39	7,65E-002
hsa-miR-3179	-1,73	7,65E-002
hsa-miR-3191	3,20	7,65E-002
hsa-miR-3189	3,52	7,84E-002
hsa-miR-671-5p	1,44	7,84E-002
hsa-miR-9	-1,51	7,99E-002
hsa-miR-431	2,27	7,99E-002

Supporting Information: Table S3

hsa-miR-18a	1,42	8,35E-002
hsa-miR-1265	-2,83	8,35E-002
hsa-miR-1286	2,19	8,35E-002
hsa-miR-4326	2,52	8,35E-002
hsa-miR-3126-5p	2,02	8,35E-002
hsa-miR-618	1,88	8,36E-002
hsa-miR-23b	-1,32	8,76E-002
hsa-miR-497	-1,35	8,76E-002
hsa-miR-3121	1,82	8,81E-002
hsa-miR-3944	6,75	8,81E-002
hsa-miR-379	1,32	8,85E-002
hsa-miR-98	1,32	8,85E-002
hsa-miR-3131	1,73	8,85E-002
hsa-miR-182	1,33	9,99E-002
hsa-miR-3913	1,56	9,99E-002
