# Supplementary material for: Differential Expression of miRNAs in Colorectal Cancer: Comparison of Paired Tumor Tissue and Adjacent Normal Mucosa Using High-Throughput Sequencing
Source: PLoS One. 2012 Apr 17;7(4):e34150. doi: 10.1371/journal.pone.0034150 (PMC3328481; doi:10.1371/journal.pone.0034150)
Supplement: Text S1 — R code for calculating differential expression. (PDF) [file pone.0034150.s007.pdf]

R Code

Using dataset processed with miRanalyzer v 0.2.
R version 2.13.0, Bioconductor version 2.8, DESeq version 1.4.1 and edgeR version 2.2.5.

```
library(DESeq)
library(edgeR)
setwd("/Users/Julian/Documents/Prosjekt/PLOS/Dataset/")

targets <- read.delim(file="Targets.txt", stringsAsFactors=FALSE)
cts <- readDGE(targets)
countsTable <- cts$counts
colnames(countsTable) <- c("T1", "T2", "T3", "T4", "T5", "T6", "T7", "T8", "N1", "N2", "N3", "N4", "N5", "N6",
"N7", "N8")
conds <- c("Ne","Ac","Ac","Ac","Ac","Ac","Ac","Ac","No","No","No","No","No","No","No","No")
cds <- newCountDataSet(countsTable, conds)
cds <- estimateSizeFactors(cds)
cds <- estimateVarianceFunctions(cds)

# Calculate dysregulated miRs using DESeq in normal mucosa (No) vs adenocarcinomas (Ac)

resNoAc <- nbinomTest(cds, "No", "Ac")
resNoAcSig <- resNoAc[resNoAc$padj<.1,]
resNoAcSig <- resNoAcSig[order(resNoAcSig$padj),]
subset(resNoAcSig, select=c(1,5,6,8))

# Calculate dysregulated miRs using DESeq in normal mucosa (No) vs neuroendocrine tumor (Ac)

resNoNe <- nbinomTest(cds, "No", "Ne")
resNoNeSig <- resNoNe[resNoNe$padj<.1,]
resNoNeSig <- resNoNeSig[order(resNoNeSig$padj),]
subset(resNoNeSig, select=c(1,5,6,8))

#Print diagnostic plot illustrating the fit of the variance function

diagForT <- varianceFitDiagnostics (cds1, "T")
smoothScatter( log10(diagForT$baseMean), log10(diagForT$baseVar) )
lines( log10(fittedBaseVar) ~ log10(baseMean), diagForT[ order(diagForT$baseMean), ], col="red" )
abline(0,1,lty=2)

#Calculate dysregulated miRs using edgeR in paired normal mucosa vs adenocarcinoma

targetsPaired <- read.delim(file="TargetsPaired.txt", stringsAsFactors=FALSE)
d <- readDGE(targetsPaired)
colnames(d) <- c("T2", "T3", "T4", "T5", "T6", "T7", "T8", "N2", "N3", "N4", "N5", "N6", "N7", "N8")
patient <- factor(c(2, 3, 4, 5, 6, 7, 8, 2, 3, 4, 5, 6, 7, 8))
design <- model.matrix(~patient + d$samples$group)
rownames(design) <- rownames(d$samples)
design[,8] <- c(1,1,1,1,1,1,1,0,0,0,0,0,0,0)
colnames(design)[8] <- "tumor"
d <- estimateGLMCommonDisp(d, design)
glmfit.d <- glmFit(d, design, dispersion = d$common.dispersion)
lrt.d <- glmLRT(d, glmfit.d, coef = 8)
options(digits = 4)
topTags(lrt.d, n=118)
```

```
#Sum of up-/downregulated miRs
sum(lrt.d$table$logFC > 0)
sum(lrt.d$table$logFC < 0)
top <- topTags(lrt.d,n=118)
sum(top$table$logFC > 0)
sum(top$table$logFC < 0)
```
